# How Far Could the Alien Boatman *Trichocorixa verticalis verticalis* Spread? Worldwide Estimation of Its Current and Future Potential Distribution

**DOI:** 10.1371/journal.pone.0059757

**Published:** 2013-03-21

**Authors:** Simone Guareschi, Cristina Coccia, David Sánchez-Fernández, José Antonio Carbonell, Josefa Velasco, Luz Boyero, Andy J. Green, Andrés Millán

**Affiliations:** 1 Department of Ecology and Hydrology, University of Murcia, Murcia, Spain; 2 Wetland Ecology Department, Doñana Biological Station (EBD-CSIC), Seville, Spain; 3 Institute of Evolutionary Biology (CSIC-Universitat Pompeu Fabra), Barcelona, Spain; 4 School of Marine and Tropical Biology, James Cook University, Townsville, Queensland, Australia; University of Florida, United States of America

## Abstract

Invasions of alien species are considered among the least reversible human impacts, with diversified effects on aquatic ecosystems. Since prevention is the most cost-effective way to avoid biodiversity loss and ecosystem problems, one challenge in ecological research is to understand the limits of the fundamental niche of the species in order to estimate how far invasive species could spread. *Trichocorixa verticalis verticalis* (*Tvv*) is a corixid (Hemiptera) originally distributed in North America, but cited as an alien species in three continents. Its impact on native communities is under study, but it is already the dominant species in several saline wetlands and represents a rare example of an aquatic alien insect. This study aims: i) to estimate areas with suitable environmental conditions for *Tvv* at a global scale, thus identifying potential new zones of invasion; and ii) to test possible changes in this global potential distribution under a climate change scenario. Potential distributions were estimated by applying a multidimensional envelope procedure based on both climatic data, obtained from observed occurrences, and thermal physiological data. Our results suggest *Tvv* may expand well beyond its current range and find inhabitable conditions in temperate areas along a wide range of latitudes, with an emphasis on coastal areas of Europe, Northern Africa, Argentina, Uruguay, Australia, New Zealand, Myanmar, India, the western boundary between USA and Canada, and areas of the Arabian Peninsula. When considering a future climatic scenario, the suitability area of *Tvv* showed only limited changes compared with the current potential distribution. These results allow detection of potential contact zones among currently colonized areas and potential areas of invasion. We also identified zones with a high level of suitability that overlap with areas recognized as global hotspots of biodiversity. Finally, we present hypotheses about possible means of spread, focusing on different geographical scales.

## Introduction

One of the most important human impacts on a wide range of ecosystems is the introduction of alien species (e.g. [1]–[4]), this being a problem of particular concern in aquatic ecosystems [5], [6] with possible impacts at different levels of organisation [7]. Alien species are a non-random subset of the aquatic biota and, although insects dominate the world’s freshwater ecosystems, they are almost unrepresented in the lists of alien species [8], [9]. In this sense, examples of the distribution, major impacts and vectors of invasive plants, fishes, mollusc and decapods are quite numerous (see [9] and references therein). However, the scientific knowledge on alien aquatic insects and their effects on biodiversity and ecosystems processes is very scarce. This is especially true for species considered to be of little importance for the economy and the general public [10].

*Trichocorixa verticalis verticalis* is one of the few strictly aquatic insects (i.e., all their life cycle stages are aquatic) that can be considered as an “alien” species because it has been moved outside of its native range, following the definitions of Rabitsch [11] and Strayer [9].

*Trichocorixa verticalis verticalis* (Fieber, 1851) (hereinafter *Tvv*) is a small corixid (Hemiptera) (<5.5 mm) originally distributed in North America and the Caribbean islands. However, this boatman has been recorded as an alien species in South Africa, New Caledonia, Morocco, Portugal and Spain, being the only water bug recognized so far as an alien species in Europe [11], [12]. The invasion of *Tvv* seems to be more widespread in the Palearctic, where it has been present in the Iberian Peninsula since at least 1997 and was first reported in Andalucía (Spain) by Günther [13]. It has since been recorded from various areas of southern Portugal [14], south-west Spain [15–17 and Authors unpublished data] and Morocco [18].

The success of this corixid as an alien species has been mainly attributed to its capacity: i) to live in brackish and saline waters in both the juvenile and adult phases [19], ii) to be passively-transported [20], and iii) to survive partial desiccation, extreme salinity or freezing in the egg stage [21]. Although this species is considered euryhaline [20], [22], it usually inhabits highly mineralized water bodies like ponds or coastal wetlands. Furthermore, *Tvv* is the only corixid found in the open sea [23]. Adults of *Tvv* also have a good ability to fly overland, which is likely to explain their colonization of closed-basin lakes in south-west Europe (e.g. numerous isolated lakes and temporary ponds throughout Andalusia [16]).

Whether this corixid is causing loss of native aquatic invertebrate populations is still partially unclear and under study, but it is the dominant hemipteran in many of the invaded sites where it is found [16], [17] and where it reproduces it is more abundant than native corixids [16]. Thus, the establishment of this species out of its native range could be considered as a threat to aquatic biodiversity, especially for local corixid species. This species also has the potential to cause major changes across food webs via trophic cascades, being one of the few predators that can survive in highly mineralized aquatic ecosystems [24].

Since prevention of invasions is the most cost-effective way to avoid biodiversity loss and nature conservation problems [25], [26], one challenge in biological invasions is to understand the limits of the fundamental niche of the species, since this information allows us to map the set of places where the species might inhabit (i.e., the potential distribution). Identification of environmentally suitable areas for invasive species can offer great opportunities for preventing or slowing invasions [27], [28]. For this purpose, ecological niche modelling has recently been used to identify the potential distributions of a number of invasive species and provide information to decision-makers (e.g. [29]–[31]). These models are designed to identify the environmental conditions in which species can maintain populations, and then to project these suitable conditions into geographical space, leading to spatial hypotheses on potential distribution (e.g. [30], [32]). These models are often coupled to climate-change models to predict how the geographic ranges of species could shift following changes in environmental conditions (e.g. [33]–[37]).

This study aims to estimate the potential distribution of *Tvv* according to the conceptual and methodological guidelines proposed by Jiménez-Valverde et al. [38]. We used complementary techniques (derived from distribution and physiology) to obtain areas of potential distribution of *Tvv* (i.e., zones with invasion risk at a global scale), taking into account both current and future climatic conditions (a climate change scenario for the year 2100).

This study represents the first attempt to estimate potential areas of invasion by *Tvv* and may be considered a useful tool to understand and prevent future invasions of this taxon in aquatic ecosystems worldwide.

## Methods

Different modelling methods may be arranged along the gradient of potential-realized distribution according to their ability to model any concept (potential distribution refers to the places where a species could live, while realized distribution refers to the places where a species actually lives; see Jiménez-Valverde et al. [39]). Since the required complexity of the modelling technique strongly depends on the precise aims, in this study we decided to use a multidimensional-envelope procedure (MDE) because it provides a picture close to the potential distribution (not the realized one; see Araújo & Peterson [40] for a review on uses and misuses of this procedure).

When estimating species’ fundamental niches, single procedures are likely to misrepresent the true range of climatic variation that those species are able to tolerate [41], and it is recommendable to consider multiple methodologies [42]. Here, the potential distribution of *Tvv* was estimated applying a multidimensional envelope procedure (MDE) based on both i) climatic data obtained from observed occurrences, and ii) thermal physiological data derived from experimental analyses. Potential distributions can be briefly considered here as the regions in which the climatic conditions are suitable for a species, according to its observed occurrences and physiological limits ([38], [43], for details).

### Estimating Potential Distribution from Occurrences (PD_O_)

We used an established procedure which maximizes the capacity to represent geographically the potential distribution of a species based only on distributional data [30], [38], [44].

### Biological and Climatic Data

**Figure 1:**
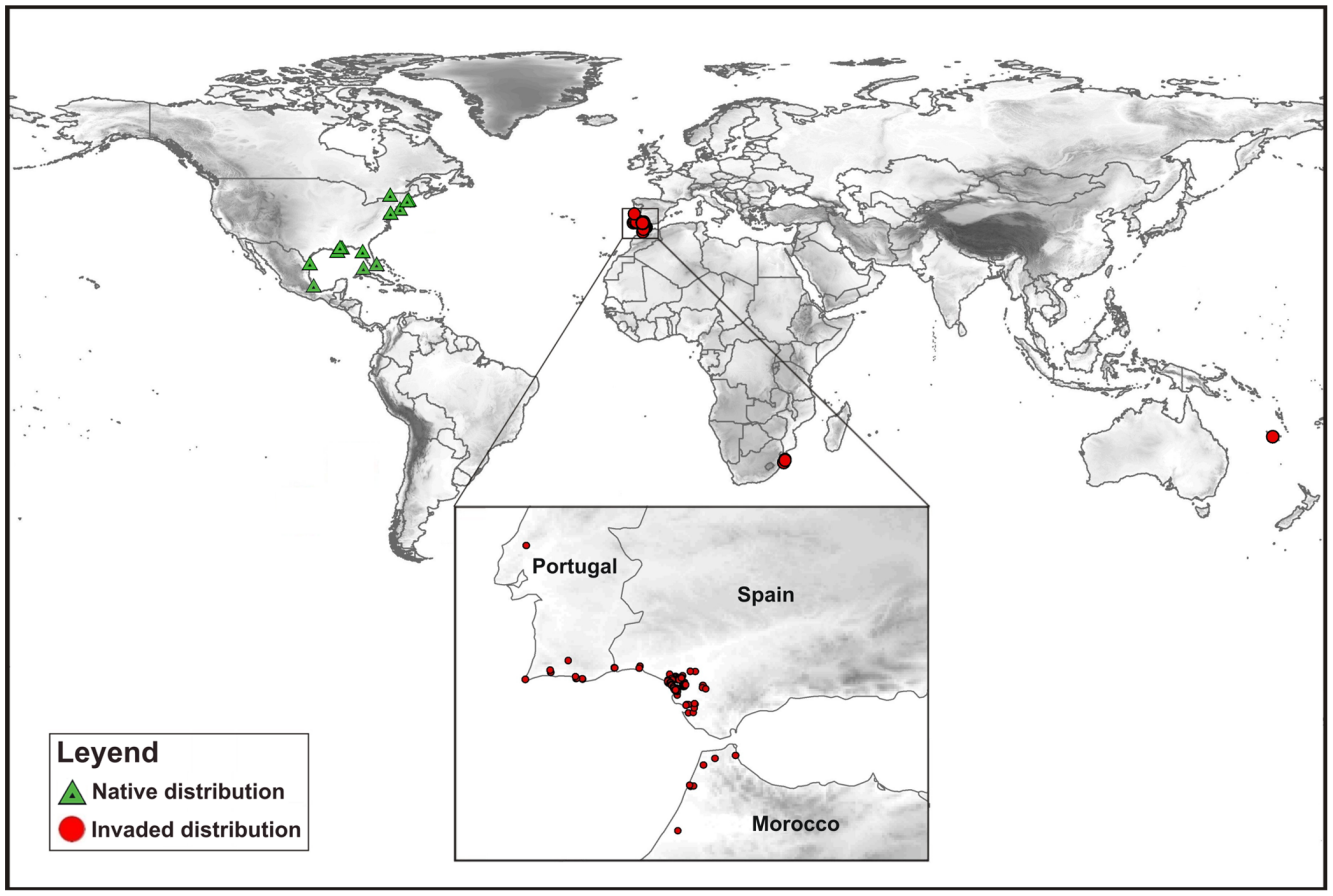
Current known distribution of *Trichocorixa verticalis verticalis*. Map of native (triangles) and invaded (circles) distribution areas of *Trichocorixa verticalis verticalis*, with a close-up of the Iberian Peninsula and Morocco.

Because species distribution models that do not incorporate global data could misrepresent potential distributions [44], we compiled all available distributional data of *Tvv* from the literature. This included published records in more than 100 years of research (1908–2011), unpublished data from sampling in invaded areas (mainly the Iberian peninsula), and data from environmental agencies’ reports and the GBIF (Global Biodiversity Information Facility, [45]). Records with taxonomic uncertainties, or doubtful or imprecise localities, were not considered in the development of predictive maps. The dataset gathered contained 152 records (species/date/locality) for *Tvv*, including both native and invaded zones (Fig. 1). As the spatial units for this study were grid cells at a resolution of 0.4°, these records were summarized in a total of thirty occurrences (0.4° grid cells).

Climatic data were obtained from WORLDCLIM, version 1.3 (http://www.worldclim.org) [46]. WORLDCLIM contains climatic data obtained by interpolation of climate station records from 1950–2000. Nineteen climatic variables were used as predictors (see Table S1 in Supporting Information). Data from all these variables were extracted at the same resolution (0.4°) as biological data.

### Selecting Relevant Variables and MDE Procedure

We used a multidimensional envelope procedure (MDE) to obtain a map with the potential distribution of *Tvv.* Firstly, and because MDE procedures are highly dependent on the number of selected predictors [47], we estimated climatic variables considered to be relevant for the species distribution. The minimum set of climatic variables needed to explain the occurrence of *Tvv* was calculated using ecological-niche factor analysis in the Biomapper package (ENFA; [48], [49]). This procedure computes uncorrelated factors that can explain both species marginality (the distance between the species optimum and the average climatic conditions in the study area) and specialization (the ratio of the ecological variance in the climate of the study area to that associated with the focal species). Factors were retained or discarded based on their eigenvalues relative to a broken-stick distribution [48]. Climatic variables selected as relevant predictors were those showing the highest correlations (factor scores >0.30) with the retained ENFA factors.

Then, the maximum and minimum scores (extreme values) for all these relevant climatic variables were calculated in all cells with observed presence of *Tvv*, selecting as suitable grid squares all those with climatic values falling within that range and designating as unsuitable all cells outside it. Distributional information from both the native range and invaded regions is recommended to improve prediction maps [38], [50]. Thus, the extreme values were used to derive a binary distributional hypothesis about the areas having climatically suitable conditions (potential distribution), assuming that recorded occurrences reflect the full spectrum of climatic conditions in which the species can survive and reproduce. Then a map with the potential distribution (PD_O_) for *Tvv* was obtained.

### Estimating Potential Distribution from Physiological Data (PD_PH_)

The potential distribution of a species can be considered to be the regions in which the climatic conditions fall within its thermal limits. Data on upper thermal limits (UTL) and lower thermal limits (LTL) were used to define *Tvv*’s thermal biology. These thermal limits were assessed by means of thermal ramping experiments (Coccia et al. unpublished data) and were obtained considering the extreme values from different combinations of temperature and conductivity during acclimatization.

These values were considered because they are the most reliable and repeatable measures of thermal limits in aquatic insects. Following the same procedure as above, suitable grid squares were considered as all those meeting two conditions: i) lower value of “maximum temperature of the warmest month” (MaxTWM) than UTL and higher value of “minimum temperature of the coldest month” (MinTCM) than LTL; i.e., the thermal values falling within the range designated as suitable by physiological experiments. In the same way, following the same procedure as above, a binary potential distribution map was derived from these physiological thermal limits (PD_PH_).

### Refining the Potential Distribution Map

**Figure 2:**
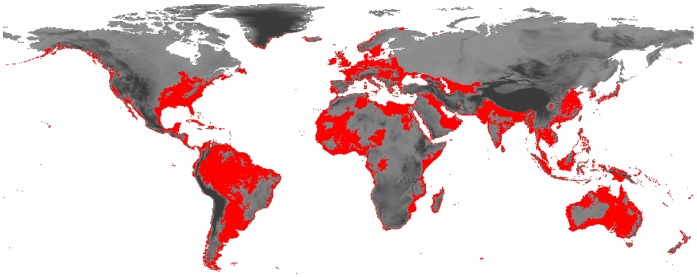
Current potential distribution. Map of worldwide potential distribution of *Trichocorixa verticalis verticalis* based on current climatic conditions.

To be conservative, we combined the potential distribution maps showing the climatically inhabitable areas for *Tvv* using both methods into a single map (PD_CL_). This new map showed all areas than can be considered as climatically suitable for *Tvv* (under current climatic conditions), considering at least one of the two procedures used (PD_CL_ = PD_O_+PD_PH_). Then, as this species mostly inhabits water bodies related with coastal environments, the PD_CL_ map was refined using altitude data as a surrogate of marine-related environments. Therefore, we removed all areas (grid cells) that presented an altitude higher than the highest altitude at which the species has been detected. We thus obtained a final potential distribution map (PD_CR_) showing the climatically suitable (under current conditions) lowland areas (Fig. 2).

### Climatic Optimum Distances

**Figure 3:**
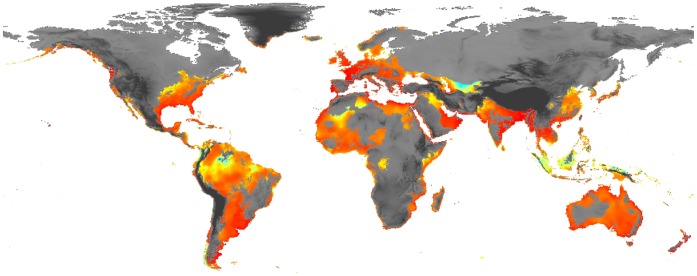
Climatic suitability within the current potential distribution. Map of worldwide current potential distribution of *Trichocorixa verticalis verticalis*. showing the climatic favorability from red (very high suitability) to light blue (very low suitability). These values were calculated applying Mahalanobis distances within the area defined in Fig. 1.

To obtain a continuous value of climatic suitability within the PD_CR_, we calculated Mahalanobis distances (a measure of multidimensional non-Euclidean distance, MD) from each cell to the mean of the hypervolume of the selected variables, with reference to the species presence records. This procedure has been widely used in spatial ecology (e.g. [51], [52]). The same predictors selected by ENFA were used to obtain MD. This process has previously been proposed as a useful tool to estimate area favourability for a species [53], and was carried out using Statistica 8.0 software [54]. Thus, the final representation of the potential distribution for *Tvv* is a map with continuous values of favourability (or climatic suitability) within its potential distribution, ranging from 0 (low suitability) to 100 (high suitability) (Fig. 3).

### Future Potential Distribution

**Figure 4:**
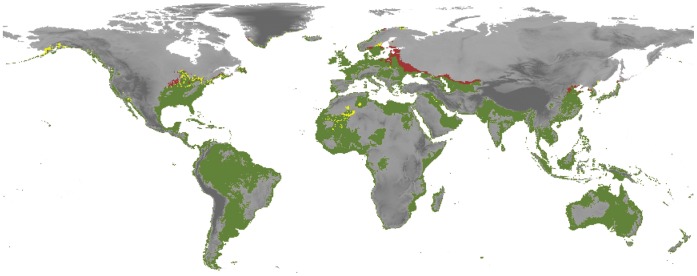
Future potential distribution of *Trichocorixa verticalis verticalis*. This map shows the worldwide future potential distribution of *Trichocorixa verticalis verticalis*. Predictions were based on the Community Climate Model scenario (CCM3) for the year 2100. The concordance between current and future periods is shown in green. Areas labeled in brown are new areas with environmental suitability for future conditions, while yellow cells represent areas where suitable climatic conditions are predicted to be lost in the future.

The extreme values found above (those obtained from both current distribution and physiology) were projected with respect to a future climate scenario, to estimate the potential dynamics of invasion risk areas through time (i.e., combining current (PD_CR_) and future (PD_F_) model outputs, see Fig. 4). Effects of climate change on the potential distribution were predicted considering a climate change Community Climate Model scenario (CCM3) for the year 2100. This prediction assumed a scenario of CO_2_ duplication in the atmosphere [55], and is approximately equivalent to the average of the current scenarios proposed by The Intergovernmental Panel on Climate Change [56]. Projected changes in aquatic habitats under climate change are based on the fact that land-based variables could be representative of climatic conditions found in inland waters, since the temperatures in these two systems are strongly correlated [57], [58], especially in shallow waterbodies in lowland areas [59] where *Tvv* lives.

## Results

### Potential Distribution Under Current Climatic Conditions

Isothermality (BIOCLIM3) and Temperature Annual Range (BIOCLIM7) were the most relevant climatic variables identified by ENFA, and therefore these variables were used in the MDE procedure. Isothermality is defined by the relationship between Mean Diurnal Range and Temperature Annual Range, and is a quantification of how large the day-to-night temperature oscillation is in comparison to the summer-to-winter oscillation (see [46]). Both variables presented negative signs, indicating that *Tvv* preferably inhabits aquatic habitats in areas with relatively constant temperature and with limited variation during the year. These climatic preferences are generally related to coastal areas, where the physical properties of the sea allow a smoothing effect of extreme temperatures.

*Tvv* presented a broad potential distribution under current climatic conditions (PD_CR_) around the world (see Fig. 2). This corixid seems to have inhabitable conditions in temperate areas, mainly in coastal areas where Isothermality and Temperature Annual Range are generally limited (Fig. 3). Nevertheless, broad areas in South America, Australia, Asia and Europe present *a priori* suitable conditions for the establishment of the species. Within this PD_CR_, the areas with higher suitability are coastal areas of Europe (including the Mediterranean islands), Turkey, Tunisia, Egypt, Myanmar, India, Argentina, Uruguay, Australia, New Zealand, the western boundary between USA and Canada, some areas of the Arabian Peninsula and the Persian Gulf (see Fig. 3).

### Potential Distribution Under Climate Change

Under the CCM3 scenario, the future climatic suitability of *Tvv* is very similar to the current potential map (Fig. 4). In general, it seems that the potential dynamics of invasion risk areas through time will be low, since almost all potential cells were maintained, with only a few additions and subtractions. In this sense, the models estimated an expansion towards higher latitudes that is visible mainly in Eastern Europe and Asia (e.g., the Baltic Republics and Ukraine), and to a lesser extent in North America. At the same time, this shift towards northern latitudes was accompanied by a reduction of suitable areas in Africa (mainly Algeria), and the loss of suitability in some cells of North America (USA and Canada).

## Discussion

### Areas of Risk Invasion and Conservation Implications

The potential distribution maps produced here represent the first attempt to estimate the global potential distribution of the alien boatman *Trichocorixa verticalis verticalis.* The most effective way to deal with introduced species, short of keeping them out, is to discover them early and attempt to eradicate or at least contain them before the extent of spread and proliferation reaches the critical threshold [60], [61]. Among our findings, one of major concern is the detection of areas highly suitable for *Tvv* in global biodiversity hotspots. Areas like the Mediterranean basin, Northern Africa, New Zealand, the Indo-Burma Region and, to a lesser extent, the Atlantic forest in South America, are particularly important given the high suitability of invasion for *Tvv*. These areas are considered important for worldwide conservation according to different global biodiversity priority templates such as the biodiversity hotspot concept [62], crisis ecoregions [63] and Global 200 biologically valuable ecoregions [64]. Our results are useful for detecting the potential connection zones between the current distribution areas (native or invaded) and other suitable areas. These zones should be kept under observation as the most likely future areas of invasion. Thus, major efforts (sampling programs, trade vigilance, biomonitoring efforts) are recommended to prevent future invasions of aquatic ecosystems in these potential areas, especially in high-risk potential contact zones (e.g. coastal wetlands in France, Italy, some areas of Northern Africa and numerous Mediterranean islands).

Western Europe has been already highlighted as a recipient area sensitive to invertebrate biological invasions [65], [66]. Strictly within the Mediterranean basin; it is interesting to note that there are so far few records of *Tvv.* To date records nearby are concentrated in Andalusian wetlands (Spain), the Algarve (Portugal) and in the Atlantic coast of Morocco, all to the west of the Strait of Gibraltar. However, samples from the Smir wetland (eastern Morocco; L’Mohdi et al. [18]) within the Mediterranean basin support the possibility that this corixid can colonize extensive areas within this basin. In Spain, Portugal and Morocco, numerous records are from protected areas such as National Parks (Doñana National Park, Southern Spain), Ramsar sites (Andalusian and Moroccan wetlands) or nature reserves (Algarve, Portugal). Biological invasions in protected areas are of global concern [67], [68] and illustrate the difficulty of managing and controlling alien species, especially invertebrates.

Invasive species coupled with climate change represent two of the most pervasive aspects of global environmental change [69]. Generally, at regional scales, a shift of species’ ranges towards higher altitudes and latitudes in accordance with their thermal preferences represents the most expected ecological impact of climatic change [70], as detected for several aquatic macroinvertebrates [71]. However, in the case of *Tvv*, potential dynamics of invasion risk areas (Fig. 4) considering future climate changes seem to be quite limited. This may be due to the low variability in the climatic conditions of the coastal areas and also because this species seems to have limited capacity to colonize water bodies in areas at high altitude. However, this restriction appears more related with habitat availability than with the apparently wide thermal tolerance of *Tvv*. Nevertheless, further experiments are needed to confirm the sensitivity response to temperature changes of this species.

### Possible Ecological Impacts and Means of Dispersal

Although insect species are extremely rare among aquatic invaders [8], *Tvv* presents traits that enable it to be an important aquatic insect invader: wide potential distribution (also in a climate change context), close relationship to coastal and transitional ecosystems which are less sensitive to drought [19]–[22], ability to exploit habitats with a high level of human impact [17], and possible capacity to be passively-transported by ships or birds (as eggs, larval and adult stages).

Humans have historically facilitated the spread of aquatic invasive species through intentional stocking, infrastructure construction, releases from aquaria and trade routes [72]. International trade has been reported to be among the most important vectors of alien species [73]. Recently, Diez et al. [74] suggested that extreme climatic events, like strong winds, large waves and high-magnitude storms, may further promote the transport, introduction and establishment of non-native species, since these events often create resource pulses that non-native species are able to utilize. Furthermore, migratory waterbirds are another plausible means by which invertebrates can colonize new areas [75].

In this sense, several studies have considered two potential means of dispersal for *Tvv*: one at an intercontinental scale (e.g., from America to Europe or Africa), and another at a more local scale (e.g., from Spain to Morocco and vice-versa or among nearby wetlands). Some studies have suggested that the presence of this corixid outside its native zone may be explained by the introduction of the fishes *Gambusia affinis*, especially in South Africa and New Caledonia [76], [77], or *Fundulus heteroclitus*, particularly in Spain (SW Europe) [14]. However, the maritime trade, which represents 90% of international trade [78], may potentially play a crucial role in dispersing *Tvv*. Ships can transport entire coastal organism assemblages across oceanic barriers and into bays, estuaries, and inland waters [79], [80]. Alien invertebrates are often transported on the surface of container ships or inside containers, as well as in ballast waters or attached to submerged objects including ballast tanks [81]–[83]. Invertebrate propagules may suffer extreme conditions during transport [84], but the wide thermal and salinity tolerance [19 and Coccia et al. unpublished data] of *Tvv*, together with its capacity to survive partial desiccation and to overwinter at the egg stage [21], [85], may allow it to survive in these environmental conditions.

Furthermore according with the BWM Convention [86] ships entering Mediterranean waters from the Atlantic Ocean (Straits of Gibraltar) should undertake ballast water exchange before entering the Mediterranean Sea. This procedure could be another option to explain the Atlantic records of *Tvv* in invaded zones (Portugal, Spain and Morocco).

Our results suggest that major maritime trade routes between commercial harbours, especially in the Atlantic (e.g., New York, Buenos Aires), as well as in Europe and Asia [87], are potential routes of *Tvv* spread. In the era of trade globalization and intensification of shipping trade, this dispersal mechanism is likely to be especially important in countries with emerging economies such as India, United Arab Emirates and China. Their rapid economic development, including an explosive growth in international trade, has already increased the potential for new introductions [88]. These new and relevant links in international trade may affect pathways for the spread of aquatic alien species, particularly euryhaline ones from coastal and transitional aquatic ecosystems, such as *Tvv*. Gaps in border controls were demonstrated to be related to alien insect invasions [89], so major efforts in terms of trade vigilance and ballast water management are recommended to prevent future spreads of *Tvv*.

Small-scale dispersal of species is mainly due to natural means such as passive transport by wind [20] or migratory waterbirds [75], [90]. Waterbirds have already been shown to disperse dipterans [91], [92], and corixid eggs can be abundant in their faeces within the *Tvv* range [93], although their viability after gut passage has not been assessed. Birds could accelerate spread across high-risk potential contact zones between currently invaded areas (e.g., Spain and Morocco) and potential areas of invasion with high level of suitability, such as coastal wetlands of France, North Africa, and Mediterranean islands. Large numbers of migratory waterbirds move through SW Spain and Morocco [94], making this flyway a potential major invasion route for *Tvv* between Africa and Europe.

Moreover, invasion of alien species is considered among the major threats to wetland ecosystems in a worldwide context, also under future global change [95]. Whether this species is contributing or not to the loss of aquatic macroinvertebrate populations in some ecosystems is still under study [17], but it is already the dominant species in several invaded saline wetlands [16]. Furthermore, environmental disturbances generally influence the invasion success of aquatic organisms [96], and *Tvv* appears to be better than native corixids at coping with human impacts and exploiting artificial wetlands [17]. In this sense, and considering the wide potential geographic range and possible capacity to be passive-transported, the establishment of this species outside of its native range may be considered as a threat to aquatic macroinvertebrate biodiversity, especially to native corixid species.

A negative impact on other invertebrates is also possible. *Tvv* is the only corixid present in several salt pan complexes in south-west Spain, and research in its native range shows it has the potential to limit the distribution of brine shrimp *Artemia*, the dominant grazer regulating phytoplankton abundance in these hypersaline systems [24]. Given the extensive overlap between the predicted distribution of *Tvv* and the current distribution of *Artemia* at a global scale [97], the spread of *Tvv* has the potential for a major impact on the distribution and abundance of brine shrimp.

### Prospects for the Future

Strong efforts are required to survey carefully the aquatic ecosystems in areas that are suitable for *Tvv*, according to our models. In many parts of the world, little attention is paid to corixids, and *Tvv* is still not present in taxonomic keys used outside North America. For this reason, it is likely that many existing populations outside the native range have so far been overlooked. Indeed, retrospective study of old samples confirmed that *Tvv* has been in the Iberia peninsula since at least 1997, but no one realized it was present prior to Günther [13]. The maps provided by this study can be used as a tool (combined with new field research) to reduce uncertainty in geographically or taxonomically questionable records coming from areas identified as suitable by our model. This could be the case of *Trichocorixa verticalis* reported without subspecies level (*Tv*) in Cuba [98] or Western Canada [99], since our maps have shown these areas to be highly suitable for *Tvv* presence (Fig. 2). Others records of *Tv* were recently reported in saline wetlands of north-western Iran [100], which our maps did not detect as a suitable area for *Tvv* invasion. These records necessarily require more research effort to clarify taxonomic doubts at the sub-species level, as correct taxonomic information is crucial for modeling studies on invasive species. Once these records (or new future records) are confirmed, they can be incorporated into our model to improve estimates of *Tvv* potential distributions.

On the other hand, genetic studies are advisable to establish whether the populations in South Africa and New Caledonia, or Europe and Africa, have a common origin, and clarify whether there have been multiple introductions from the native range. Furthermore, genetic studies are also required to clarify the separation of *Tv* into subspecies, and to address possible differences in their capacities to be invasive.

## Supporting Information

Table S1 **Climatic variables.** Set of Bioclimatic variables considered. Variables were derived from the monthly data. A quarter is a period of three months (1/4 of the year).
(DOC)
