# Supplementary material for: How Far Could the Alien Boatman Trichocorixa verticalis verticalis Spread? Worldwide Estimation of Its Current and Future Potential Distribution
Source: PLoS One. 2013 Mar 21;8(3):e59757. doi: 10.1371/journal.pone.0059757 (PMC3605378; doi:10.1371/journal.pone.0059757)
Supplement: Table S1 — Climatic variables. Set of Bioclimatic variables considered. Variables were derived from the monthly data. A quarter is a period of three months (1/4 of the year). (DOC) [file pone.0059757.s001.doc]

**Supporting Information**

**Table S1. Climatic variables**

Set of Bioclimatic variables considered. Variables were derived from the monthly data. A quarter is a period of three months (1/4 of the year).

| Code | Bioclimatic Variables |
| --- | --- |
| BIO1 | Annual Mean Temperature |
| BIO2 | Mean Diurnal Range (Mean of monthly (max temp - min temp)) |
| BIO3 | Isothermality (BIO2/BIO7) (* 100) |
| BIO4 | Temperature Seasonality (standard deviation *100) |
| BIO5 | Max Temperature of Warmest Month |
| BIO6 | Min Temperature of Coldest Month |
| BIO7 | Temperature Annual Range (BIO5-BIO6) |
| BIO8 | Mean Temperature of Wettest Quarter |
| BIO9 | Mean Temperature of Driest Quarter |
| BIO10 | Mean Temperature of Warmest Quarter |
| BIO11 | Mean Temperature of Coldest Quarter |
| BIO12 | Annual Precipitation |
| BIO13 | Precipitation of Wettest Month |
| BIO14 | Precipitation of Driest Month |
| BIO15 | Precipitation Seasonality (Coefficient of Variation) |
| BIO16 | Precipitation of Wettest Quarter |
| BIO17 | Precipitation of Driest Quarter |
| BIO18 | Precipitation of Warmest Quarter |
| BIO19 | Precipitation of Coldest Quarter |
|  | |
